# Sulfatinib, a novel kinase inhibitor, in patients with advanced solid tumors: results from a phase I study

**DOI:** 10.18632/oncotarget.14942

**Published:** 2017-02-01

**Authors:** Jian Ming Xu, Yan Wang, Yu Ling Chen, Ru Jia, Jie Li, Ji Fang Gong, Jing Li, Chuan Qi, Ye Hua, Cui Rong Tan, Jian Wang, Ke Li, Yang Sai, Feng Zhou, Yong Xin Ren, Wei Guo Qing, Hong Jia, Wei Guo Su, Lin Shen

**Affiliations:** ^1^ Department of Gastrointestinal Oncology, The Affiliated Hospital Cancer Center (The 307th Hospital of Chinese People's Liberation Army), Academy of Military Medical Sciences, Beijing, China; ^2^ Department of Gastrointestinal Oncology, Peking University Cancer Hospital, Beijing, China; ^3^ Clinical and Regulatory Department, Hutchison MediPharma Limited, Shanghai, China; ^4^ Drug Metabolism and Pharmacokinetic Department, Hutchison MediPharma Limited, Shanghai, China; ^5^ Oncology Department, Hutchison MediPharma Limited, Shanghai, China; ^6^ Chemistry Department, Hutchison MediPharma Limited, Shanghai, China

**Keywords:** phase I clinical trial, neuroendocrine tumor, solid tumor, sulfatinib, tyrosine kinase inhibitor

## Abstract

Sulfatinib is a small molecule kinase inhibitor that targets tumor angiogenesis and immune modulation. This phase I study (NCT02133157) investigated the safety, pharmacokinetic characteristics, and preliminary anti-tumor activity of sulfatinib in patients with advanced solid tumors. The study included a dose-escalation phase (50-350 mg/day, 28-day cycle) with a Fibonacci (3+3) design, and a tumor-specific expansion phase investigating the tumor response to treatment. Two sulfatinib formulations were assessed: formulation 1 (5, 25, and 50 mg capsules) and formulation 2 (50 and 200 mg capsules). Seventy-seven Chinese patients received oral sulfatinib; the maximum tolerated dose was not reached. Dose-limiting toxicities included abnormal hepatic function and coagulation tests, and upper gastrointestinal hemorrhage. The most common treatment-related adverse events were proteinuria, hypertension and diarrhea. Among 34 patients receiving sulfatinib formulation 2, one patient with hepatocellular carcinoma and eight with neuroendocrine tumors exhibited a partial response; 15 had stable disease. The objective response rate was 26.5% (9/34) and the disease control rate was 70.6% (24/34). Pharmacokinetic, safety, and efficacy data supported continuous oral administration of sulfatinib at 300 mg as the recommended phase II dose. Sulfatinib exhibited an acceptable safety profile and encouraging antitumor activity in patients with advanced solid tumors, particularly neuroendocrine tumors.

## INTRODUCTION

Vascular endothelial growth factor (VEGF)- and fibroblast growth factor (FGF)-mediated pathways play key roles in tumor angiogenesis [1, 2]. VEGF and FGF secretion by tumor cells promotes rapid proliferation and packing of endothelial cells, which leads to the formation of excessive, coarsely packed blood vessels [3]. These blood vessels supply oxygen and nutrients to the tumor and promote tumor cell leakage into the circulation, resulting in increased tumor growth and a risk of metastasis [3]. While VEGF receptor (VEGFR)-targeted therapies are important in the management of several cancer types, many patients exhibit no or limited respond to treatment, due in part to tumor cell resistance through alternative molecular pathways [4].

In response to anti-VEGF therapies, some tumors can increase FGF secretion to stimulate endothelial cell proliferation, promote tumor angiogenesis, and bypass VEGF signaling pathways [4, 5]. Evidence also suggests that VEGFR, FGF receptors (FGFRs), and colony stimulating factor 1 receptor (CSF1R) promote tumor immune evasion. VEGF secreted by tumors can activate VEGFR signaling pathways in T cells; this leads to programmed cell death protein 1 (PD-1) receptor overexpression, which decreases T cell anti-tumor activity [6]. FGFR and CSF1R also appear to induce tumor-associated macrophage proliferation and differentiation, thereby promoting tumor immune evasion [7].

Targeting multiple kinases to simultaneously block VEGFR-, FGFR-, and CSF1R-mediated pathways may be a more effective method of preventing tumor angiogenesis and tumor immune evasion, and therefore represents an attractive anti-cancer therapy approach. Sulfatinib (HMPL012) is a potent small molecule tyrosine kinase inhibitor of VEGFR 1, 2, and 3, FGFR 1, and CSF1R [8], and has demonstrated selectivity in a broad kinase screening (Table 1). The aims of this phase I clinical study in patients with advanced solid tumors were to determine the sulfatinib maximum tolerated dose (MTD) and recommended dose for further phase II investigations. The study was designed to investigate the safety, pharmacokinetics (PK), and tumor response of sulfatinib.

**Table 1:** Sulfatinib kinase selectivity profile

Kinase	IC_50_ (μM)
VEGFR 1	0.002
VEGFR 2	0.024
VEGFR 3	0.001
FGFR1	0.015
CSF1R	0.004
TrkB	0.041
FLT3	0.067
278 other kinases	>0.150

FLT3: fms-related tyrosine kinase 3; IC_50_: half maximal inhibitory concentration; TrkB: tropomyosin receptor kinase B.

## RESULTS

### Patient baseline characteristics

**Figure 1:**
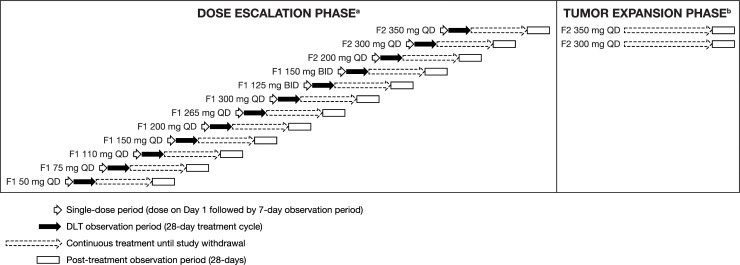
Study design ^a^Sulfatinib dose was escalated (until MTD was met) according to a modified Fibonacci 3+3 protocol. Each patient received the assigned dose for the study duration. ^b^The tumor expansion phase was initiated following determination of the recommended phase II dose based on the results of the dose-escalation phase.

**Figure 2:**
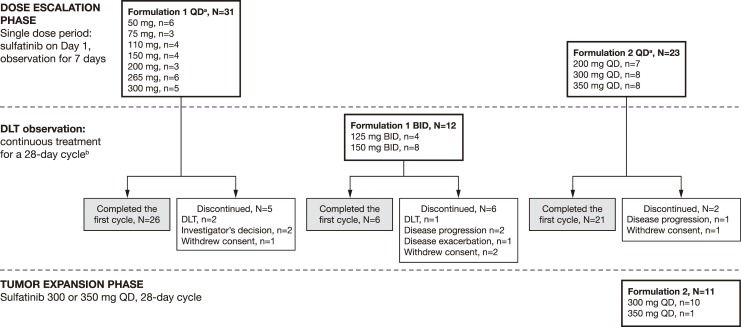
Patient configuration ^a^At enrolment, patients were assigned a dose sequentially according to the Fibonacci 3+3 dose-escalation design. Patients received that dose for the study duration. ^b^Patients who completed the DLT observation phase could remain on treatment at their original dose until disease progression or any other withdrawal criteria were met.

Seventy-seven Chinese patients were enrolled in 12 dose cohorts between April 2010 and September 2014, and followed up until July 2015 (Figure 1). The first 43 patients received sulfatinib formulation 1 and the remaining 34 received formulation 2 (Figure 2). Patient baseline demographic and clinical characteristics are summarized in Table 2.

**Table 2:** Patient baseline demographic and clinical characteristics

Characteristic	Formulation 1 (N=43)	Formulation 2 (N=34)
**Median (range) age, years**	52.7 (23.5–69.9)	56.0 (23.4–73.2)
**Gender, n (%)**		
Male	27 (62.8)	24 (70.6)
Female	16 (37.2)	10 (29.4)
**Tumor type, n (%)**		
Colorectal carcinoma	9 (20.9)	0
Hepatocellular carcinoma	8 (18.6)	9 (26.5)
Stromal tumor	8 (18.6)	1 (2.9)
NET (grade 1/2)^a^	7 (16.3)	21 (61.8)
Non-small cell lung cancer	2 (4.7)	0
Renal cell carcinoma	2 (4.7)	0
Other	7 (16.3)	3 (8.8)
**ECOG performance status, n (%)**		
0	10 (23.3)	4 (11.8)
1	29 (67.4)	30 (88.2)
2	4 (9.3)	0
**Median (range) time since diagnosis, years**	1.9 (0.1–11.2)	0.8 (0.0–6.8)
**Previous anti-tumor systemic therapy, n (%)**		
Yes	33 (76.7)	20 (58.8)
No	10 (23.3)	14 (41.2)

^a^NET pathology grading was categorized according to Ki67 index and tumor cell mitotic rate. Grade 1 and 2 tumors were also reported as well-differentiated NETs.

### Sulfatinib exposure, dose escalation, and dose-limiting toxicities

Sixty-six patients were enrolled in the dose-escalation phase; of these, 53 (80.3%) completed the first treatment cycle. Discontinuation reasons included disease progression (n=3) or deterioration (n=1; combined total n=4, 6.1%), consent withdrawal (n=4, 6.1%), dose-limiting toxicities (DLTs) (n=3, 4.5%), and investigator's decision (n=2, 3.0%).

In the dose-escalation phase, 43 patients received continuous treatment with sulfatinib formulation 1 at 50 mg, 75 mg, 110 mg, 150 mg, 200 mg, 265 mg, and 300 mg doses once daily (QD), and 125 mg and 150 mg twice daily (BID) (Figure 2). Median treatment duration with formulation 1 was 32.5 days (range 2–269 days). Three patients experienced DLTs (one Grade 3 abnormal coagulation with sulfatinib 50 mg QD; one Grade 3 upper gastrointestinal [GI] hemorrhage with sulfatinib 265 mg QD; one Grade 3 abnormal hepatic function with sulfatinib 150 mg BID). Twenty-three patients received sulfatinib formulation 2 during the dose-escalation phase at doses of 200 mg, 300 mg, and 350 mg QD (Figure 2). In addition, 11 patients were treated with 300 mg or 350 mg QD sulfatinib formulation 2 during the expansion phase. Median treatment duration with formulation 2 was 147.5 days (range 9–644 days). One patient receiving sulfatinib formulation 2 at 200 mg QD experienced a DLT (Grade 3 increased alanine transaminase [ALT]/aspartate aminotransferase [AST]).

MTD was not reached with sulfatinib doses of up to 350 mg QD (formulation 2). The initial plan was to escalate the formulation 2 dose up to 400 mg QD; however, drug exposure (AUC, area under the concentration-time curve; C_max_, peak concentration) at 350 mg QD was no higher than that at 300 mg QD. Based on the available PK, safety, and efficacy data, the investigator and sponsor agreed there would be no further dose escalation even though MTD had not been reached.

### Safety

Forty-two patients (97.7%) receiving formulation 1 experienced one or more adverse events (AEs). The most common treatment-related AEs (TRAEs) (occurring in ≥10% patients) were: asthenia; increased blood bilirubin; protein present in urine; increased AST; diarrhea; increased blood pressure; hypomagnesemia; increased white blood cell count; abdominal pain; blood in urine; hypocalcemia; hypokalemia; and pyrexia. Grade 3 TRAE incidence was 25.6%, with increased AST (n=2, 4.7%) and decreased hemoglobin (n=2, 4.7%) most common. Other Grade 3 TRAEs (abdominal pain, diarrhea, upper GI hemorrhage, gastric dysfunction, abnormal hepatic function, gastroenteritis, infection, increased ALT, increased blood pressure, abnormal coagulation test, hypokalemia, hypoproteinemia, nephrotic syndrome, and pelvi-ureteric obstruction) each occurred in a single patient (2.3%). There were no Grade 4 or 5 TRAEs. There were five serious AEs (SAEs) with formulation 1, three of which were considered by the investigator as possibly related to the study drug: a Grade 3 nephrotic syndrome and a Grade 3 upper GI hemorrhage, both in patients receiving 265 mg QD; and a Grade 3 hepatic function abnormality in a patient receiving 150 mg BID. These three patients discontinued sulfatinib and received supportive care. The patient with a Grade 3 hepatic function abnormality died 23 days after the last study dose; disease progression was considered the primary cause of death.

All patients treated with formulation 2 experienced at least one AE. The most common TRAEs of any grade (occurring in ≥10% of patients) are summarized in Table 3. Overall Grade 3 and 4 TRAE incidence was 47.1%, and the most common was proteinuria (14.7%; Table 3). No Grade 5 AEs were reported. Eight SAEs were reported for formulation 2, two of which were considered by the investigator to be possibly related to the study drug: a Grade 3 upper GI hemorrhage in the 300 mg QD dose cohort; and a case of Grade 3 acute pancreatitis in the 350 mg QD dose cohort. Most SAEs resolved, with the exception of an intra-abdominal hemorrhage (unrelated to the study drug) and an intervertebral disc protrusion (unlikely related to the study drug).

**Table 3:** TRAEs occurring in ≥10% of patients treated with sulfatinib formulation 2 (N=34)

System organ class Preferred term, n(%)	200 mg QD, N=7		300 mg QD, N=18		350 mg QD, N=9		Total, N=34	
	Any grade	Grade 3/4	Any grade	Grade 3/4	Any grade	Grade 3/4	Any grade	Grade 3/4
**Blood and lymphatic system disorders**								
Anemia	0	0	2 (11.1)	0	2 (22.2)	1 (11.1)	4 (11.8)	1 (2.9)
**Cardiac disorders**								
Sinus bradycardia	0	0	4 (22.2)	0	1 (11.1)	0	5 (14.7)	0
**Gastrointestinal disorders**								
Diarrhea	3 (42.9)	0	8 (44.4)	1 (5.6)	8 (88.9)	1 (11.1)	19 (55.9)	2 (5.9)
Abdominal discomfort	2 (28.6)	0	3 (16.7)	0	6 (66.7)	0	11 (32.4)	0
Nausea	3 (42.9)	0	4 (22.2)	0	2 (22.2)	0	9 (26.5)	0
Abdominal distention	1 (14.3)	0	4 (22.2)	0	4 (44.4)	0	9 (26.5)	0
**General disorders and administration site conditions**								
Asthenia	2 (28.6)	0	4 (22.2)	0	6 (66.7)	1 (11.1)	12 (35.3)	1 (2.9)
Face edema	0	0	2 (11.1)	1 (5.6)	5 (55.6)	0	7 (20.6)	1 (2.9)
Edema peripheral	0	0	2 (11.1)	1 (5.6)	3 (33.3)	0	5 (14.7)	1 (2.9)
**Investigations**								
Blood pressure increased	0	0	10 (55.6)	2 (11.1)	4 (44.4)	0	14 (41.2)	2 (5.9)
Blood TSH increased	1 (14.3)	0	7 (38.9)	0	5 (55.6)	0	13 (38.2)	0
Blood bilirubin increased	2 (28.6)	0	8 (44.4)	1 (5.6)	3 (33.3)	0	13 (38.2)	1 (2.9)
AST increased	2 (28.6)	1 (14.3)	6 (33.3)	1 (5.6)	4 (44.4)	0	12 (35.3)	2 (5.9)
Blood triglycerides increased	0	0	5 (27.8)	0	7 (77.8)	0	12 (35.3)	0
WBC count decreased	1 (14.3)	0	5 (27.8)	0	5 (55.6)	0	11 (32.4)	0
Neutrophil count decreased	2 (28.6)	0	4 (22.2)	1 (5.6)	4 (44.4)	0	10 (29.4)	1 (2.9)
Blood albumin decreased	1 (14.3)	0	8 (44.4)	0	1 (11.1)	0	10 (29.4)	0
Platelet count decreased	2 (28.6)	0	3 (16.7)	0	5 (55.6)	2 (22.2)	10 (29.4)	2 (5.9)
Abnormal ECG T-wave	1 (14.3)	0	6 (33.3)	0	2 (22.2)	0	9 (26.5)	0
Blood uric acid increased	1 (14.3)	0	4 (22.2)	1 (5.6)	4 (44.4)	0	9 (26.5)	1 (2.9)
ALT increased	2 (28.6)	1 (14.3)	2 (11.1)	0	2 (22.2)	0	6 (17.6)	1 (2.9)
Blood creatinine increased	1 (14.3)	0	0	0	5 (55.6)	0	6 (17.6)	0
Thyroxine free decreased	0	0	5 (27.8)	0	1 (11.1)	0	6 (17.6)	0
Hemoglobin decreased	1 (14.3)	0	1 (5.6)	1 (5.6)	3 (33.3)	1 (11.1)	5 (14.7)	2 (5.9)
Thyroid function test abnormal	1 (14.3)	0	1 (5.6)	0	2 (22.2)	0	4 (11.8)	0
**Metabolism and nutrition disorders**								
Hypoproteinemia	0	0	10 (55.6)	0	7 (77.8)	0	17 (50.0)	0
Hypocalcemia	0	0	7 (38.9)	0	5 (55.6)	0	12 (35.3)	0
Decreased appetite	0	0	6 (33.3)	0	5 (55.6)	0	11 (32.4)	0
Hypokalemia	2 (28.6)	0	4 (22.2)	0	2 (22.2)	1 (11.1)	8 (23.5)	1 (2.9)
Hypertriglyceridemia	0	0	4 (22.2)	0	4 (44.4)	0	8 (23.5)	0
Hyperuricemia	0	0	4 (22.2)	1 (5.6)	3 (33.3)	0	7 (20.6)	1 (2.9)
Hypophosphatemia	2 (28.6)	0	3 (16.7)	2 (11.1)	1 (11.1)	0	6 (17.6)	2 (5.9)
Hyponatremia	0	0	3 (16.7)	0	1 (11.1)	0	4 (11.8)	0
Hypercholesterolemia	0	0	0	0	4 (44.4)	0	4 (11.8)	0
**Musculoskeletal and connective tissue disorders**								
Back pain	1 (14.3)	0	3 (16.7)	0	1 (11.1)	0	5 (14.7)	0
**Nervous system disorders**								
Dizziness	1 (14.3)	0	2 (11.1)	0	3 (33.3)	0	6 (17.6)	0
**Renal and urinary disorders**								
Proteinuria	2 (28.6)	0	11 (61.1)	3 (16.7)	7 (77.8)	2 (22.2)	20 (58.8)	5 (14.7)
**Vascular disorders**								
Hypertension	0	0	5 (27.8)	1 (5.6)	2 (22.2)	0	7 (20.6)	1 (2.9)

TRAE: treatment-related adverse event; TSH: thyroid stimulating hormone; WBC: white blood cell; ECG: electrocardiogram.

### Pharmacokinetic profile

Sixty-eight patients were eligible for the steady-state PK assessment, including 40 patients who received sulfatinib formulation 1 and 28 who received formulation 2 (Table 4). For formulation 1, following QD administration for 14 days within the dose range of 50–265 mg, sulfatinib exposure (indicated by AUC) generally increased dose-proportionally. There was no AUC increase when the dose increased from 265 mg to 300 mg. Median time to C_max_ (T_max_) ranged from 1.8 to 3.5 hours. Both C_max_ and AUC showed high inter-subject variability with coefficient of variation (CV%) up to 69.5% for C_max_ (75 mg group) and 68.8% for AUC (300 mg group). Following BID administration for 14 days, mean AUC values were similar at 125 and 150 mg (1977 versus 1952 ng·hour/mL) with a CV% up to 64.8%.

**Table 4:** Sulfatinib pharmacokinetic parameters on day 14 of continuous dosing

PK parameter	Sulfatinib formulation 1 dose								
	50 mg QD (N=5)	75 mg QD (N=3)	110 mg QD (N=4)	150 mg QD (N=3)	200 mg QD (N=3)	265 mg QD (N=6)	300 mg QD (N=5)	125 mg BID (N=3)	150 mg BID (N=8)
Mean C_max_ (CV%), ng/mL	84 (56.4)	123 (69.5)	262 (35.3)	293 (35.7)	370 (20.3)	498 (66.9)	546 (67.3)	267 (52.1)	248 (65.9)
Median T_max_ (range), hour	1.8 (1.0–4.0)	2.0 (1.0–4.0)	1.5 (0–4.0)	1.0 (1.0–1.0)	3.0 (1.0–4.0)	3.5 (1.0–4.0)	2.8 (1.0–4.0)	3.0 (1.0–4.0)	3.4 (1.0–8.0)
Mean AUC^a^ (CV%), ng·hour/mL	654 (51.2)	964 (32.3)	2579 (28.2)	2308 (48.8)	3314 (13.1)	5958 (65.2)	5403 (68.8)	1977 (59.8)	1952 (64.8)
**PK parameter**	**Sulfatinib formulation 2 dose**								
	**200 mg QD (N=6)**			**300 mg QD (N=14)**			**350 mg QD (N=8)**		
Mean C_max_ (CV%), ng/mL	549 (73.1)			625 (54.6)			655 (32.5)		
Median T_max_ (range), hour	1.0 (1.0–2.0)			2.0 (1.0–4.1)			2.0 (2.0–4.0)		
Mean AUC^a^ (CV%), ng·hour/mL	4273 (55.0)			5116 (50.4)			5289 (37.6)		

^a^AUC_0-24_ for QD and AUC_0-12_ for BID.

For formulation 2, following consecutive QD administration for 14 days, mean AUC at 200, 300, and 350 mg was 4273, 5116, and 5289 ng·hour/mL, respectively, indicating that sulfatinib exposure was similar at 300 mg and 350 mg, but higher than that at 200 mg. The inter-subject variability was high, as indicated by a CV% of up to 55% for AUC and 73.1% for C_max_. Median T_max_ ranged from 1.0 to 2.0 hours for the test dose levels.

### Clinical response

Among 43 patients treated with sulfatinib formulation 1, 12 patients were not evaluable for efficacy, either because they did not have a post-treatment tumor assessment, or because they exhibited SD at the first post-treatment assessment (week 4), but no additional assessment to demonstrate that the SD continued for a minimum of six weeks from baseline. Of the 31 patients evaluable by Response Evaluation Criteria in Solid Tumors (RECIST) Version 1.0, none achieved complete response (CR) or partial response (PR). Eight patients had stable disease (SD) and 23 had progressive disease (PD).

**Figure 3:**
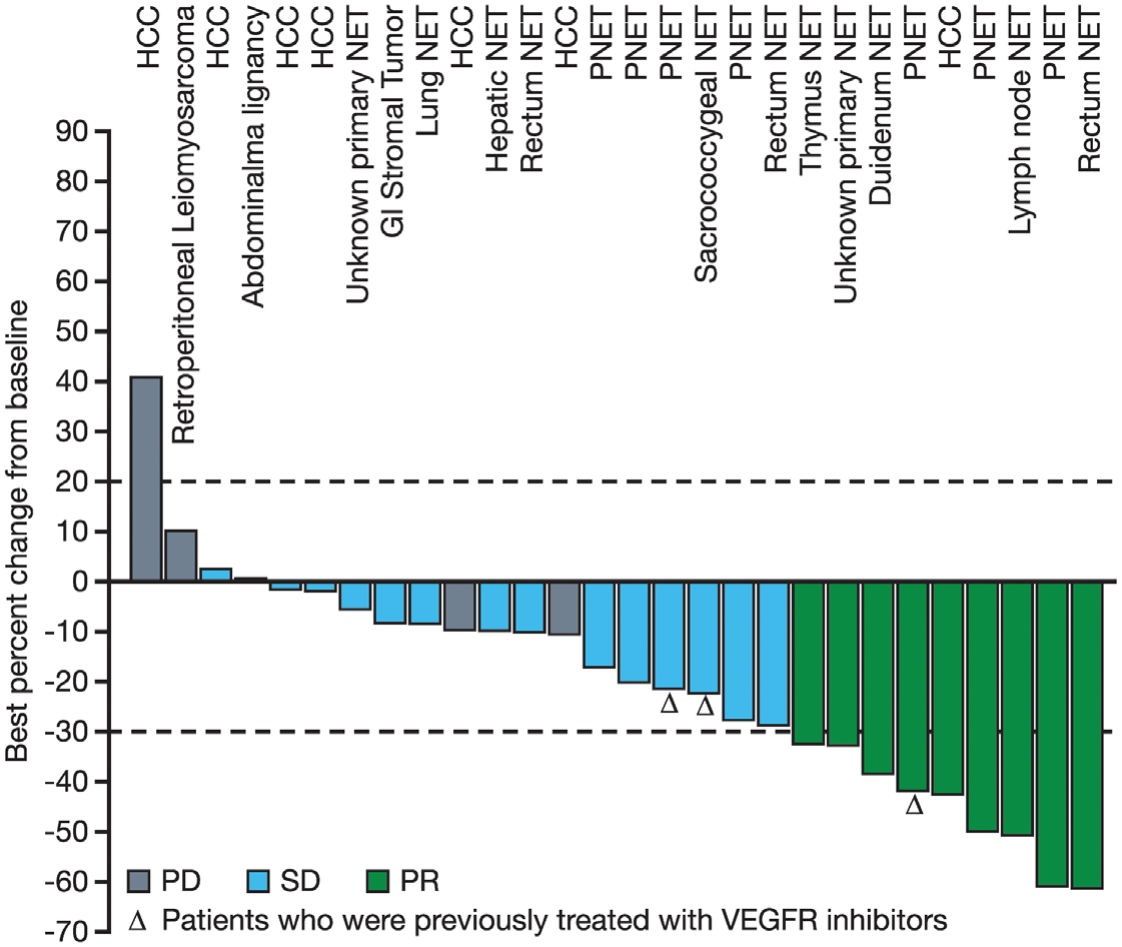
Best percent change in tumor size (sum of diameter of target lesions) compared with baseline for efficacy-evaluable patients treated with sulfatinib formulation 2 (N=28) PNET: pancreatic neuroendocrine tumor.

Among 34 patients treated with formulation 2, six were not evaluable for response due to early discontinuation without adequate post-treatment tumor evaluation. Of the 28 patients evaluable by RECIST criteria, nine achieved PR (Figure 3), including one patient with hepatocellular carcinoma receiving sulfatinib 200 mg QD, and eight with neuroendocrine tumors (NETs) receiving sulfatinib 300 or 350 mg QD. There were 15 patients with SD (10 with NETs, three with hepatocellular carcinoma, one with GI stromal tumors, and one with an abdominal malignancy) and four patients with PD.

The objective response rate (ORR) for all 77 patients was 11.7% (9/77) and disease control rate (DCR) was 41.6% (32/77). The ORR of patients treated with sulfatinib formulation 2 was 26.5% (9/34) and DCR was 70.6% (24/34). Tumor response rate was similar among patients receiving higher formulation 2 doses. In the 300 mg QD cohort, ORR was 27.8% (5/18) and DCR was 66.7% (12/18), while in the 350 mg QD cohort, ORR was 33.3% (3/9) and DCR was 77.8% (7/9).

**Figure 4:**
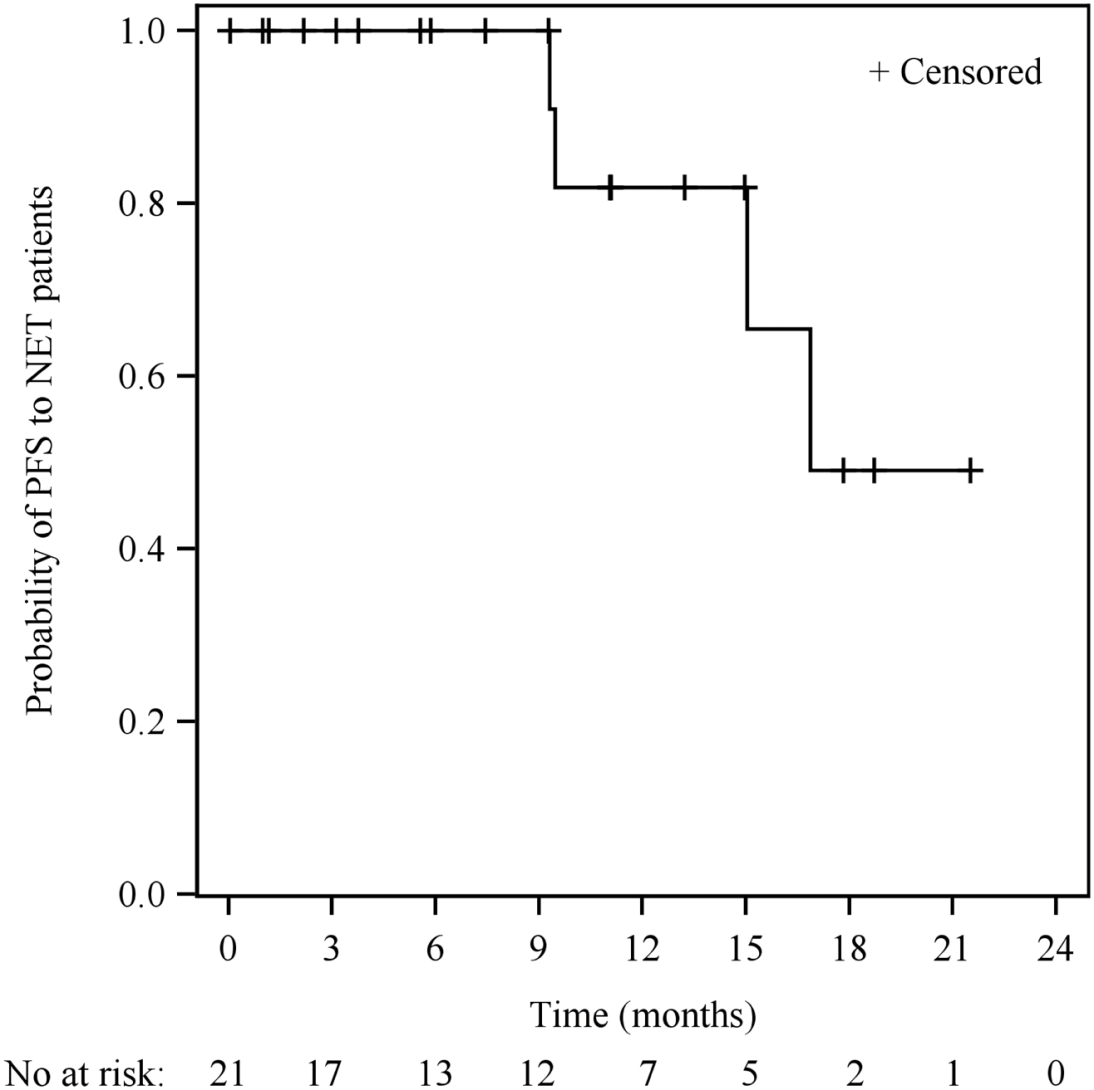
Kaplan-Meier survival curve of PFS in NET patients treated with sulfatinib formulation 2 (N=21)

There were 21 patients with well-differentiated NETs (grade 1 or 2) treated with sulfatinib formulation 2 at 200-350 mg QD. Within this subgroup, eight patients achieved PR, 10 achieved SD, and three were not evaluable for response, with an ORR of 38.1% (8/21) and DCR of 85.7% (18/21). Tumor origins of the eight NET patients who achieved PR were: pancreas (n=3); duodenum (n=1); rectum (n=1); thymus (n=1); and unknown (n=2). Median time to response (TTR) was 3.0 months (range 1.3–10.2 months). Median duration of response (DoR) was 15.7 months (95% confidence interval [CI]: 3.8–not reached [NR]). Median progression free survival (PFS) was 16.9 months (95% CI: 9.5–NR) (Figure 4). Notably, three NET patients who had previously been treated, but progressed on VEGFR kinase inhibitors (such as sunitinib or famitinib), obtained clinical benefit from sulfatinib, with two patients achieving SD and one achieving PR (treatment duration from 5.1 to 11.6 months).

## DISCUSSION

Sulfatinib, a potent oral kinase inhibitor targeting VEGFR (1, 2, 3), FGFR1, and CSF1R with good selectivity, has demonstrated anti-angiogenic and anti-tumor activity in preclinical studies (Hutchison MediPharma, unpublished data). This first-in-human, phase I study investigated the safety, PK characteristics, and preliminary anti-tumor activity of sulfatinib in patients with advanced solid tumors.

MTD was not reached within sulfatinib doses of 50-350 mg QD and 125-150 mg BID, and sulfatinib appeared to be generally well tolerated. Most AEs were mild to moderate and could be managed through dose adjustment or supportive care. The most commonly reported AEs, including proteinuria, hypertension, and diarrhea, were consistent with AEs seen with VEGFR tyrosine kinase inhibitors [9–12].

Sulfatinib demonstrated promising anti-tumor activity against advanced solid tumors in our study. Nine out of the 77 patients had a confirmed PR and 23 had sustainable SD. Clinical efficacy was observed with sulfatinib formulation 2 at doses from 200 mg QD, with nine patients achieving a PR and 15 achieving SD. PK analyses demonstrated that sulfatinib was rapidly absorbed and drug exposure (AUC and C_max_) generally increased with dose escalation. Drug exposure began to plateau at a dose of 265 mg for formulation 1 and 300 mg for formulation 2, suggesting potential absorption saturation. Inter-patient variation in drug exposure was moderate to high across all dose levels for formulation 1, and appeared moderately improved for formulation 2.

Sulfatinib was well tolerated up to 350 mg QD. Drug exposure (AUC) did not increase with a dose increase from 300 to 350 mg QD (formulation 2). This finding suggested potential absorption saturation and that further dose escalation would not achieve an increase in drug exposure. However, there was a higher incidence of Grade ≥3 AEs with the 350 mg dose compared to 300 mg during the first cycle of the continuous dose period. Although sample size was small, the two dose cohorts demonstrated comparable anti-tumor activity. Together, the PK, safety, and efficacy data support the selection of 300 mg QD as the recommended phase II dose.

Unresectable or metastatic NET is a rare and life-threatening disease with limited treatment options [13–15]. Median survival varies from several months to a few years depending on primary tumor site [16]. In recent years, only two targeted therapies have been approved by the United States Food and Drug Administration for treatment of advanced well-differentiated NETs: sunitinib, a multi-kinase inhibitor mainly targeting angiogenesis, and everolimus, an oral mammalian target of rapamycin (mTOR) inhibitor. In a phase III sunitinib trial in pancreatic NET patients, ORR was 9.3% with PFS of 11.0 months [11], although sunitinib failed to demonstrate effectiveness in extra-pancreatic NET patients. In phase III trials, median PFS of pancreatic NET or GI and lung NET patients treated with everolimus was both 11.0 months, while ORR was only 5% and 2%, respectively [17, 18].

The present study included 21 patients with advanced NETs treated with sulfatinib formulation 2. Robust clinical activity was demonstrated in these cases, with an ORR of 38.1%, DCR of 85.7%, and a median PFS of 16.9 months (95% CI: 9.5–NR). Notably, anti-tumor activity was demonstrated by sulfatinib in NET patients regardless of tumor origin, and also in three patients who had previously failed prior VEGFR inhibitor treatment. This suggests that sulfatinib, which simultaneously targets tumor angiogenesis and immune evasion, may provide clinical benefit for NET patients [7, 19]. The efficacy analysis should be interpreted with caution due to the small sample size, and the non-randomized, open-label study design with no comparator. Nevertheless, the preliminary results of this study provide sufficient support to warrant further sulfatinib anti-tumor efficacy evaluation. Investigations into the sulfatinib mechanism of anti-tumor activity are ongoing, both in preclinical and clinical settings, and may provide more support for the use of sulfatinib in advanced solid tumors.

In summary, sulfatinib has demonstrated promising anti-tumor activity in patients with advanced solid tumors, especially NETs, and was generally well tolerated. Our results will be further validated in a phase Ib/II study in NET patients (NCT02267967) and in two randomized, double-blind, placebo-controlled, multicenter phase III trials: one in patients with pancreatic NETs (NCT02589821) and one in patients with extra-pancreatic NET (NCT02588170).

## MATERIALS AND METHODS

### Patients

Patients were recruited from the Affiliated Hospital Cancer Center (the 307th Hospital of Chinese People's Liberation Army), Academy of Military Medical Sciences, Beijing, China, and Peking University Cancer Hospital, Beijing, China. Patients with recurrent and/or metastatic malignant solid tumors were eligible for this study if they had shown disease progression after standard therapy or were unable to receive standard therapy. Eligible patients were 18–75 years old, with an Eastern Cooperative Oncology Group (ECOG) performance status ≤2, and a life expectancy of >3 months. Patients with uncontrolled brain metastases were excluded. Pre-treatment evaluations included: a physical examination; ECOG performance status; laboratory tests for renal, liver, and metabolic functions; cardiac function (electrocardiogram and ultrasonic cardiogram); and a pregnancy test for female patients of childbearing age.

### Study design and dose administration

The primary objectives of this open-label, first-in-human phase I study (NCT02133157) were to determine MTD and the phase II dose of sulfatinib, and to evaluate the safety of sulfatinib in patients with advanced solid tumors. The secondary and exploratory objectives included evaluation of sulfatinib PK and preliminary anti-tumor activity. The study consisted of a dose-escalation phase (split into a single-dose period and continuous-dose period) and a tumor-specific expansion phase. Two sulfatinib formulations were used during the study: formulation 1 (5, 25, and 50 mg capsules) and formulation 2 (50 and 200 mg capsules). The study was conducted in accordance with the Good Clinical Practice Guidelines of the International Council for Harmonization of Technical Requirements for Pharmaceuticals for Human Use. The protocol was approved by each participating institution's ethics review board. All patients provided written informed consent.

During the dose-escalation phase, patients were given a single dose of sulfatinib and monitored for AEs for seven days. If no clinically significant toxicities were observed, patients could enter the 28-day DLT observation phase where they received sulfatinib continuously for 28 days. DLTs were assessed at the end of the 28-day period. If no patients experienced a DLT during the 28-day period, the dose was escalated. After completion of the DLT observation phase, patients could continue treatment at their current dose (if they were judged by the investigator to be benefiting from treatment) until any of the withdrawal criteria (investigator's decision that withdrawal was in the patient's best interest, intolerable toxicity, disease progression) were met.

The study used a modified Fibonacci 3+3 dose-escalation design with at least three evaluable patients treated with each dose. The MTD was defined as the maximum dose at which no more than one of six evaluable patients experienced a DLT during the first 28-day treatment period (cycle). For each dose, if no patients experienced a DLT during the treatment cycle, the dose was escalated for the next dose cohort. If one of the first three patients treated at a dose experienced a DLT, three additional patients were added to expand the cohort. If two or more of the first three or overall six patients experienced a DLT, the MTD was considered exceeded and the previous lower dose would be re-assessed to determine the MTD. Patients who completed the first treatment cycle and fulfilled ≥75% of the planned accumulating dose, or who experienced a DLT any time during the first treatment cycle, were considered as DLT-evaluable patients. Dose reductions were not permitted in the first treatment cycle.

After the MTD and recommended phase II dose were established and preliminary efficacy data from the dose-escalation phase had demonstrated an effective dose range (a PR had been observed), the study was expanded to investigate tumor response at the identified doses (300 mg and 350 mg QD [formulation 2]) in patients with advanced solid tumors. Patients with NETs were enrolled with priority as preliminary efficacy was shown in these tumor types in the dose-escalation stage.

### Endpoints and analyses

Safety and tolerability were assessed in all patients who received at least one dose of sulfatinib throughout the study. AEs were recorded throughout the study. All AEs were coded by organ system using preferred terms as per the Medical Dictionary for Regulatory Activities (MedDRA) Version 17.0, and were graded using the National Cancer Institute Common Terminology Criteria for Adverse Events (NCI CTCAE) Version 3.0. AE frequency, severity, and relationship to study drug were summarized and tabulated, together with SAEs or deaths. TRAEs were defined as AEs that were considered by the investigators to be possibly, probably or definitely related to the study treatment.

DLTs were defined as any of the following toxicities occurring in the first continuous-dosing treatment cycle (day 1-28) of the dose-escalation phase: any non-hematologic toxicity ≥ Grade 3 in severity, except for fatigue, nausea, vomiting, diarrhea, constipation, pain, and hypertension, which were considered DLTs if they were ≥ Grade 3 after adequate treatment; hematologic toxicities, including Grade 4 decreases in white blood cell count, platelet count or hemoglobin; Grade 3 febrile neutropenia; and Grade 3 decreases in platelets with hemorrhage tendency. Physical examinations, ECOG performance status, and laboratory tests were obtained in the single-dose screening period at day 1, and during the continuous-dose period were collected weekly in the first treatment cycle, every two weeks in the second treatment cycle, and every four weeks in the third treatment cycle onwards.

For the assessment of sulfatinib PK at steady state, plasma samples were collected from each patient prior to treatment, and at 1, 2, 4, 8, 12, and 24 hours on day 14 for QD cohorts or at 1, 4, 8, and 12 hours following the first dose on day 14 for BID cohorts. PK parameters analyzed were AUC, C_max_, and T_max_. Phoenix WinNonlin 6.3 software was used to analyze descriptive statistics of concentration data and PK parameters, and to plot plasma concentration-time curves. AUC was calculated using the linear trapezoidal area method.

Tumor response (an exploratory endpoint) was assessed according to RECIST Version 1.0 and measured at baseline, at the end of every treatment cycle in the first four cycles, and every other cycle thereafter. Patients with an initial assessment of CR or PR had this result confirmed by a repeat tumor assessment at least four weeks later. Calculations of the following parameters were made: ORR (CR + PR); SD defined as ≥1 assessment of SD at least six weeks after study entry; DCR (CR + PR + SD). TTR, DoR and PFS were analyzed in the subgroup of patients with NET.
